# Improvement in Stroke-induced Motor Dysfunction by Music-supported Therapy: A Systematic Review and Meta-analysis

**DOI:** 10.1038/srep38521

**Published:** 2016-12-05

**Authors:** Yingshi Zhang, Jiayi Cai, Yaqiong Zhang, Tianshu Ren, Mingyi Zhao, Qingchun Zhao

**Affiliations:** 1 School of Life Sciences and Biopharmaceutics, Shenyang Pharmaceutical University, Shenyang, 110016, P.R. China; 2 Department of Pharmacy, General Hospital of Shenyang Military Area Command, Shenyang, 110840, P.R. China

## Abstract

To conduct a meta-analysis of clinical trials that examined the effect of music-supported therapy on stroke-induced motor dysfunction, comprehensive literature searches of PubMed, Embase and the Cochrane Library from their inception to April 2016 were performed. A total of 10 studies (13 analyses, 358 subjects) were included; all had acceptable quality according to PEDro scale score. The baseline differences between the two groups were confirmed to be comparable. Compared with the control group, the standardized mean difference of 9-Hole Peg Test was 0.28 (−0.01, 0.57), 0.64 (0.31, 0.97) in Box and Block Test, 0.47 (0.08, 0.87) in Arm Paresis Score and 0.35 (−0.04, 0.75) in Action Research Arm Test for upper-limb motor function, 0.11 (−0.24, 0.46) in Berg Balance Scale score, 0.09 (−0.36, 0.54) in Fugl-Meyer Assessment score, 0.30 (−0.15, 0.74) in Wolf Motor Function Test, 0.30 (−0.15, 0.74) in Wolf Motor Function time, 0.65 (0.14, 1.16) in Stride length and 0.62 (0.01, 1.24) in Gait Velocity for total motor function, and 1.75 (0.94, 2.56) in Frontal Assessment Battery score for executive function. There was evidence of a positive effect of music-supported therapy, supporting its use for the treatment of stroke-induced motor dysfunction. This study was registered at PRESPERO (CRD42016037106).

Stroke is a multifaceted and complicated condition. Stroke disease is one of the major causes of long-term disability and one of the leading causes of death worldwide^1^,^2^. The time frequency and functional source analysis of the signals facilitate the quantification of the functional changes occurring in the brain in association with motor tasks after stroke and the detection of damage to neuro-motor functioning^3^. The personal burden of being a stroke survivor is often devastating and has major consequences for the patient’s quality of life^4^. Rehabilitation of upper-limb motor dysfunction and total motor dysfunction have been revealed to improve the quality of life of patients after stroke^5^ and are safe and effective methods for restoring social and occupational functioning.

Motor dysfunction therapy relies on both pharmacological^6^ and non-pharmacological treatments^7^. Currently, pharmacological therapy is essentially symptomatic and does not have a satisfactory impact on symptoms related to the progression of neurodegenerative diseases. Therefore, several health institutions recommend the development of non-pharmacological complementary interventions as a first-line treatment. For example, intensive motor therapy can improve important motor functions. However, the effectiveness of standard physiotherapeutic approaches in stroke rehabilitation has been found to be limited^8^. In the human brain, one of the most powerful sources of auditory stimulation is provided by music^9^. As a result, more attention has been given to the effectiveness of non-pharmacological approaches in dysfunction therapy, including a growing interest in music therapy and music-based stimulation^10^.

The power of music and its nonverbal nature make it an effective medium of communication when language is diminished or abolished, though the curative effect of music is still uncertain. Music easily elicits movement, stimulating interactions between perception and action systems^11^. Thus, music-making may be an effective way to induce plastic changes in the motor system. Music-supported therapy is a prospective new series of therapy programs, and comprehensive research suggests that it could be useful because of its promotion of relaxation and of cognitive and motor improvement in post-stroke rehabilitation^12^. Therefore, music-supported therapy has been developed with the aim of improving motor recovery after stroke. The definition of music-supported therapy is not only hearing the music but also singing and playing rhythm and percussion instruments and is based on four principles: (i) massive repetition and exercising of simple finger and arm movements; (ii) auditory-motor coupling and integration and reinforcement of motor effects due to immediate auditory feedback; (iii) shaping and adapting the training according to individual progress; and (iv) emotion-motivation effects due to the playfulness and emotional impact of music and the acquisition of a new skill^13^. Music-supported therapy may involve, for example, rhythmic auditory stimulation^14^, the use of a MusicGlove^15^ or listening to CDs^16^. However, the differences between these music-supported techniques have not been comprehensively considered.

Music-supported therapy has been shown to be effective in post-stroke rehabilitation of motor function in some clinical trials^14^,^15^,^16^,^17^,^18^,^19^,^20^,^21^,^22^,^23^. However, little research has focused on the potential therapeutic mechanisms by which music-supported therapy improves the motor functions of post-stroke patients. Although many researchers suggest that improvement induced by music-supported therapy is due to the combined effects of intensive repetitive practice and musical stimulation^21^, evidence to support these propositions has been unavailable. To explore the isolated effect of music further, we designed a systematic review on the effect of music-supported therapy on the recovery of upper-limb motor function and total motor function after stroke. No previous reviews have provided a comprehensive overview with meta-analyses.

## Results

### Baseline characteristics

**Figure 1:**
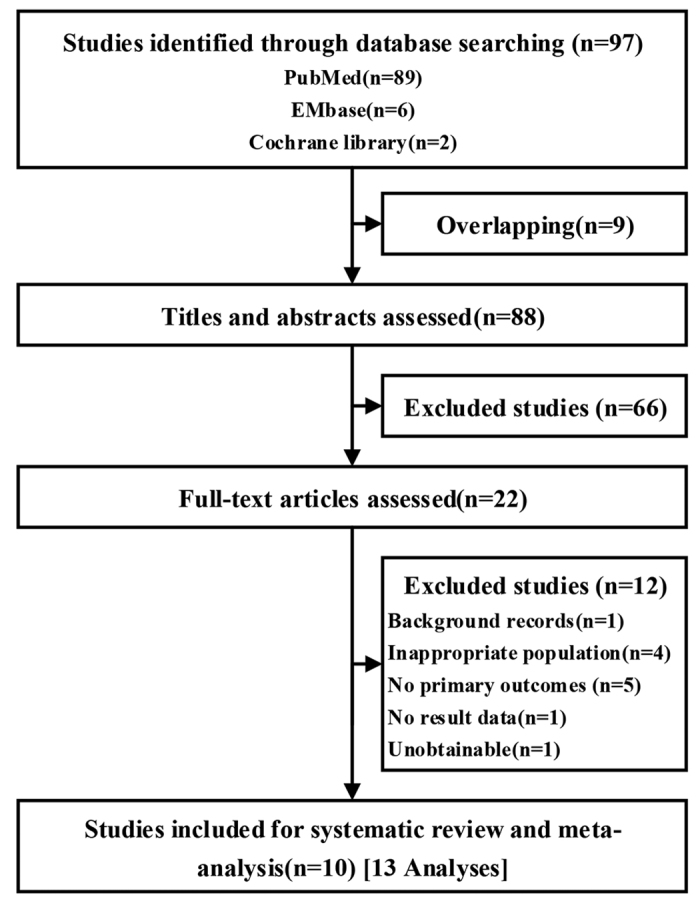
Flow of studies through the review process for systematic review and meta-analysis.

In total, 10 eligible studies (13 analyses, 358 participants)^14^,^15^,^16^,^17^,^18^,^19^,^20^,^21^,^22^,^23^ were identified and incorporated into the systematic review and meta-analysis (Fig. 1). Of these 10 studies, 7 were randomized controlled trials (RCT), 2 of them as controlled clinical trials (CCT), and 1 was a randomized crossover trial (RCT/crossover). All included studies assessed motor function as an outcome. Summaries of the characteristics of the included trials appear in Table 1 and Table S1. The studies were conducted in a wide range of countries and continents, the publication dates ranged from 2003 to 2016, and the included studies had between 12 and 62 subjects. Table S1 summarizes the detailed characteristics of the music-supported therapy group and the control group. The statistics showed that the two groups had similar characteristics in terms of age, stroke type, time post stroke, and position (left/right). There was some evidence of a difference in gender between the two groups; however, the difference was small. In particular, baseline characteristics were comparable (Table 1).

Researchers generally trained the subjects with music in an interactive way, meaning that subjects not only passively listened to music on a CD player but also sang and played rhythm and percussion instruments. Comparator descriptions varied and included active control (such as tabletop exercises or audio books) and usual therapy. The duration of music-supported therapy varied between 2 weeks to 6 months.

### Methodological quality

The assessments of study quality are presented in Table 1 and Table S2. The result of the PEDro scale score showed that all of our included studies had acceptable quality. Most of the studies mentioned were blinded, but a few of them were described as double-blinded (blinding to participants and therapists). Furthermore, the quality of the studies was positively correlated with the designs of the trials: RCT and RCT/crossover were superior to CCT.

### Efficacy of music-supported therapy on upper-limb motor function

**Figure 2:**
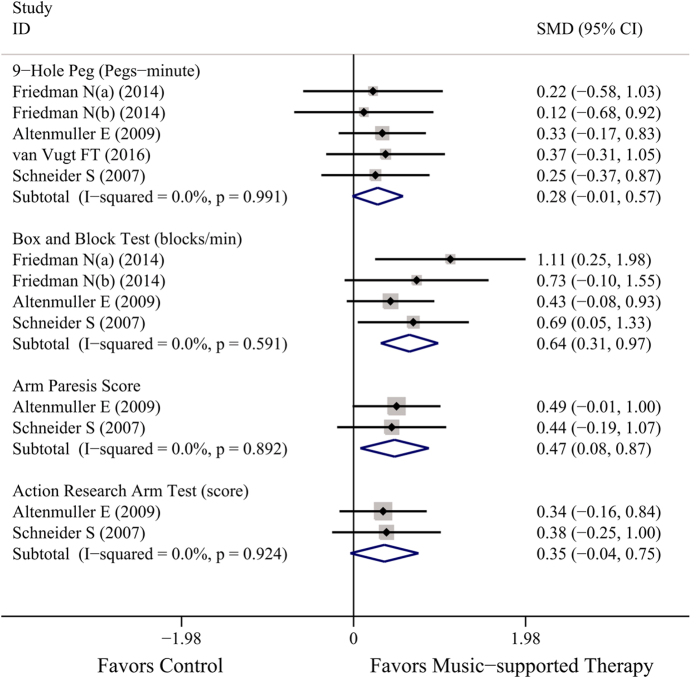
Overall efficacy of music-supported therapy for upper-limb motor function.

The included studies were suitable for meta-analysis of upper-limb motor function (Fig. 2), and all of the data were tested after the therapy ended. These studies contributed to four separate subanalyses, each with different types of evaluated measures.

For studies that evaluated the effect of music-supported therapy using the 9-Hole Peg Test (9HPT, Pegs-minute)^15^,^17^,^19^,^21^, the result (SMD = 0.29, 95% CI: −0.01~0.57) revealed no significant difference between the two arms with low heterogeneity (*P* = 0.991, *I*^2^ = 0%) among the included studies. However, the confidence interval does not exclude the possibility of a positive effect meaning that music may have a positive trend to adjust motor function.

Four analyses (3 studies)^15^,^19^,^21^ reported the effect of the Box and Block Test (BBT, blocks/min), and the merged result showed not only a significant positive effect of music-supported therapy (SMD = 0.64, 95% CI: 0.31~0.97) but also no heterogeneity (*P* = 0.591, *I*^2^ = 0%) among the included studies.

Two trials^19^,^21^ also presentedthe Arm Paresis Score (APS) and the Action Research Arm Test (ARAT) for patients; however, we were only able to find a significant difference in the ARS test (SMD = 0.47, 95% CI: 0.08~0.87) and no heterogeneity among the two types of tests (*I*^2^ = 0%).

### Efficacy of music-supported therapy for total motor function

**Figure 3:**
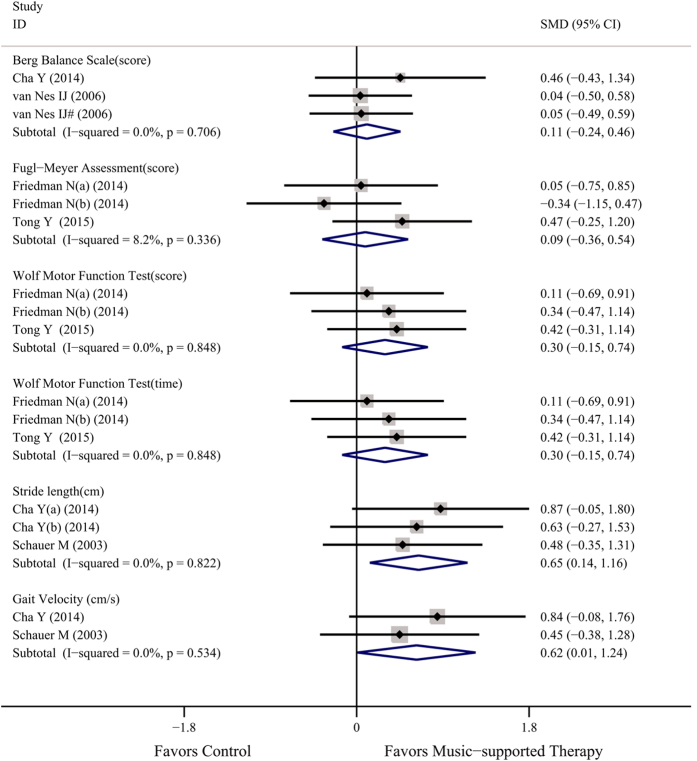
Overall efficacy of music-supported therapy for total motor function.

The included studies were suitable for the meta-analysis of total motor function (Fig. 3), and the results at the end of treatment and follow-up were merged for analysis. These studies contributed to six separate sub-analyses, each with different types of evaluated measures.

Two studies (3 analyses) reported the Berg Balance Scale (BBS) score^14^,^22^, each providing two results, one at the end of treatment and one at follow-up. There was no heterogeneity between the trials (*P* = 0.706, *I*^2^ = 0%). In a random effects meta-analysis, the SMD was 0.11 (95% CI: −0.24~0.46), suggesting that music-supported therapy might be beneficial to improve total motor function, although no significant difference was found between the two groups.

Two studies^15^,^18^ presented the Fugl-Meyer assessment score (FMA), the Wolf Motor Function Test score (WMFT) and the Wolf Motor Function Test time, with no heterogeneity found in any of the 3 evaluated measures (*P* > 0.05, *I*^2^ < 10%). The results of the FMA (SMD = 0.09, 95% CI: −0.36~0.54), the WMFT score (SMD = 0.30, 95% CI: −0.15~0.74) and the WMFT time (SMD = 0.30, 95% CI: −0.15~0.74) revealed that music-supported therapy group achieved better curative effects than the control group.

Total motor function could also be reported by stride length (cm) from 3 therapy-ending data^14^,^23^, and the merged results favored the music-supported therapy group (SMD = 0.65, 95% CI: 0.14~1.16) and showed no heterogeneity among studies (*P* = 0.822, *I*^2^ = 0%). These two studies also reported Gait Velocity (cm/s). No significant heterogeneity could be found (*P* = 0.534, *I*^2^ = 0%). The result (SMD = 0.62, 95% CI: 0.01~1.24) revealed that a significant difference existed between the two groups.

### Efficacy of music-supported therapy for executive function

**Figure 4:**
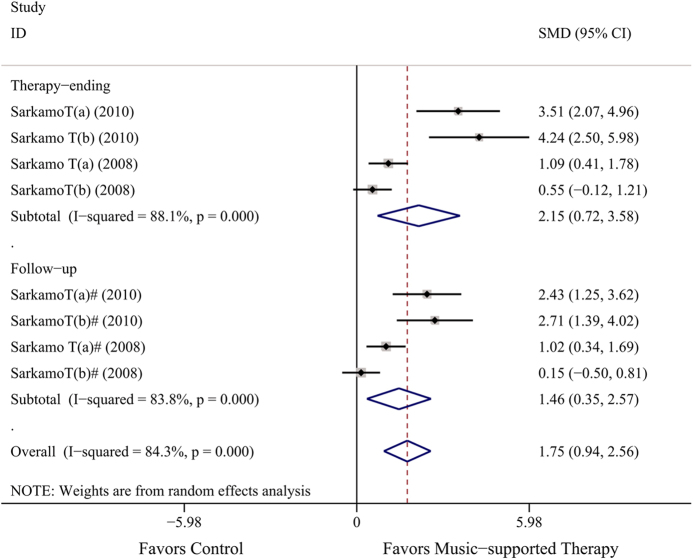
Overall efficacy of music-supported therapy for executive function.

The overall effect on executive function was evaluated by the Frontal Assessment Battery (FAB) score, which was 1.75 (SMD, 95% CI: 0.94~2.56) from 4 therapy-ending data and 4 follow-up data^15^,^20^, revealing significant differences between the two groups. This means that music-supported therapy could improve executive function, although heterogeneity among studies was found (*P* = 0.000, *I*^2^ = 84.3%). Because of intensive heterogeneity, we did a subgroup analysis by time of data evaluated. In the therapy-ending group, the result of SMD was 2.35 (95% CI: 0.72~3.58; *P* = 0.000, *I*^2^ = 88.1%), and the SMD was 1.46 in the follow-up group (95% CI: 0.35~2.57; *P* = 0.000, *I*^2^ = 83.8%, Fig. 4).

## Discussion

Our meta-analysis suggests that music-supported therapy has a positive effect on motor function as evaluated by the following instruments: the 9-Hole Peg Test, the Box and Block Test, the Arm Paresis Score and the Action Research Arm Test for upper limb motor function; the Berg Balance Scale, the Fugl-Meyer Assessment, the Wolf Motor Function Test, and Stride length and Gait Velocity for total motor function; and the Frontal Assessment Battery for executive function. This finding was based on a comprehensive systematic review including 10 studies (13 analyses), and nearly 400 subjects. Most trials suggested that music-supported therapy was associated with improvements in motor function. However, some outcomes did not reach statistical significance, and heterogeneity existed in others (Figs 2, 3 and 4).

For upper-limb function, four measures showed the same trend, although only two were significant (BBT and APS), and the differences in effect size were modest. The BBT (Fig. 2), which quantifies important skills—grasp and transport—and is simple and quick to administer, is more objective than the 9-HPT, APS and ARAT because it depends less on the semi-subjective rating of the evaluator. The BBT has excellent reliability and is correlated with APS and ARAT. For the six measures of total motor function, well-established clinical measures only demonstrated a positive direction of music-supported therapy, such as the BBS, while the sensitivity of the WMFT was better than that of the FMA. Moreover, significant differences appeared in stride length and gait velocity (cm/s) (Fig. 3). For executive function, both time points were significant with high heterogeneity (Fig. 4).

To determine the source of homogeneity, we used subgroup analysis on the outcome of the Frontal Assessment Battery. However, no significant differences existed between the two subgroups of the evaluation time, meaning that the leading cause of homogeneity was not found. In our meta-analysis, two of these evaluation instruments included the data at the assessment time of follow-up. The result demonstrated that, whether at the end of treatment or during follow-up after a period of time, music-supported therapy was effective. Moreover, results may be better at the end of treatment (Figs 3 and 4). Though we only used eleven instruments for assessment, more indicators in our included fundamental research are reported, such as MMNm ampliture^16^, SS-QoL score^14^, etc. Although these indicators have not been included in our meta-analysis because few analyses used them (less than two), they also showed a favorable effect of music-supported therapy. Although lacking data, the included trials were compliant with a good standard of quality, and we believe that this meta-analysis is the most comprehensive systematic review so far to investigate the use of music-supported training in stroke-induced motor dysfunction therapy. No adverse effects were reported in our included fundamental studies.

Our conclusion by meta-analysis should be verified. A published article confirmed that music-supported therapy is a viable intervention to improve motor function in chronic stroke patients^13^, which is consistent with the results of our meta-analysis. Moreover, the results of one study demonstrate the feasibility of rhythmic auditory stimulation to enhance gait training, which warrants further investigation of the protocol to demonstrate the effects of rhythmic auditory stimulation in stroke rehabilitation^24^. Furthermore, a case-report study assessed technology-aided intervention programs, which involved activating music for two post-coma men who had re-acquired consciousness, and participants enjoyed the intervention sessions with the programs and reported that the programs had beneficial effects for them^25^. The significance of that study is not just in supporting music-supported therapy but also in showing that technology-aided intervention programs that included music also had a positive effect. Not only our included population should be considered. Jamali S.^26^ and Amengual J.L.^27^ in their experiment used healthy subjects as a comparator, and their conclusions supported the use of music-supported therapy for chronic stroke patients. While Tan L.F.^28^ researched only healthy participants, the music-supported therapy group also experienced the expected effects.

The present meta-analysis has several limitations. We undertook this systematic review with a comprehensive search strategy, and although there were no data and language restrictions, it was impossible to include all published and unpublished literature, especially the unpublished literature. Furthermore, positive results are easy to publish, but negative results are not likely to leave the laboratory. Another limitation was that many of the included studies had very small sample sizes (the average sample size was less than 40), which means that many of our included studies may have lacked test powers to detect differences between the intervention group and control group. An additional limitation of many outcomes was their extensive heterogeneity, which indicated substantial variability in the outcomes of the included studies, although this was often because of the presence of baseline differences (Table 1) and anticipated differences in trial design, populations and country. For example, if the difference in gender is too large, it may lead to heterogeneity. Moreover, lack of randomization, inadequate randomization and allocation concealment were more likely to lead to heterogeneity. Subgroup analyses generally did not substantially explain and reduce the heterogeneity: we used a random effects model that takes heterogeneity into account, and the results could be explained as reflecting the average result across the group of studies. Finally, publication bias could not be excluded because the funnel plots were not able to assess publication bias in our meta-analysis due to the limited number of trials.

The beneficial effects of music-supported therapy on participants are consistent with expectations and perceptions of music. Several possible and potential mechanisms could help to explain the effects of music training on neurodegenerative symptoms. Several potential mechanisms could help to explain the effects of music-supported therapy. For example, these mechanisms may involve structural and functional neural reorganization in the brain following injury^29^. The understanding of the brain’s plastic properties has led to the emergence of new approaches in stroke rehabilitation^30^. However, the mechanisms underlying successful musical neurodegenerative dysfunction rehabilitation are not well understood. The discovery of the clinical effectiveness of rhythmic motor entertainment also brought into focus for the first time that the structural elements of music have enormous potential in clinical applications to retrain the injured brain^31^.

Previous narrative reviews of non-pharmacological therapy have reported positive results. Generally speaking, a previous meta-analysis^32^ based on stroke patients had similar trends as the results we obtained, meaning that non-pharmacological therapy will be important to pursue in future clinical practice. Music is a non-pharmacological, non-invasive, non-adverse reaction and inexpensive intervention training that can be delivered easily and successfully. Further clinical trials of music-supported therapy should include large sample sizes, be robust, and be randomized to confirm the effect of music-supported therapy, particularly on patient-relevant or disease-specific outcomes. Further studies should ensure that the appropriate methods are used for randomization, blinding and intent-to-treat. Further trials should assess outcomes using standardized or prescribed measures at similar time points. Analyses of individual data would be valuable for further exploration. More normative studies will be used for further meta-analysis.

In summary, there was evidence of a positive effect of music-supported therapy, supporting its use for the treatment of motor dysfunction. On a local scale, patients with stroke-induced motor dysfunction could be encouraged to undertake music-supported therapy.

## Methods

We followed the standards set by the systematic review and meta-analyses (PRISMA)^33^ statement, and our study was conducted according to the protocol registered with PROSPERO (Number CRD42016037106)^34^.

### Eligibility criteria

We included randomized controlled trials, controlled clinical trials, and randomized crossover trials that compared music-supported therapy to no music therapy or to usual care of patients with stroke-induced motor dysfunction. We considered trials including motor function tests except those that did not use motor function as the leading indicator. Patients diagnosed with any type of stroke by each individual study were accepted.

We searched PubMed, Embase and Cochrane Library for clinical trials published up to April 2016. There were no language restrictions for the search. We combined both MeSH and free text for identifying relevant literature.

### Data collection, extraction and quality assessment

Two investigators (ZYS and CJY) examined study eligibility. Both independently extracted and tabulated data from the studies on a standardized data extraction form, and disagreements were resolved through consensus or referral to a third reviewer (ZMY or ZQC). Discrepancies and unobtainable data were resolved by group discussion of at least three investigators. Randomized controlled trails (RCT), controlled clinical trials (CCT) and randomized crossover trials (RCT/crossover, before-after studies without control groups) were eligible for this meta-analysis.

We extracted baseline information on publication, year, country, study design, participants (n, age, male%), stroke type, position (left/right), delivery, etc. from each study. The design of every individual study was also used as baseline information, including intervention method, intervention frequency and duration, and outcome assessment time.

We assessed the quality of the included studies with the Physiotherapy Evidence Database (PEDro) scale score^35^. The PEDro is an 11-item scale to assess the quality of clinical trials. If the answer of the first item was “NO”, the study was excluded from meta-analysis. When the PEDro score is >4 (max score was 10), the study is considered high quality. Different trial designs result in a different score (RCT, CCT, other and unclear), thus affecting the final scores. Data extraction and quality assessment were performed independently by two investigators and adjudication by the third when required.

### Outcome measures

The outcome was motor function, which included upper limb motor function and total motor function; meta-analysis was suitable for this outcome, although we used various tests to obtain it. The 9-Hole Peg Text (9HPT, Pegs-minute)^21^, Box and Block Test (BBT)^36^, Arm Paresis Score (APS) and Action Research Arm Test (ARAT)^37^ are used for upper-limb motor function assessment. Furthermore, the Berg Balance Scale (BBS)^38^, Fugl—Meyer assessment (FMA)^39^, Wolf motor function test (WMFT)^40^, and Stride length (SL) and gait velocity (cm/s)^41^ are used for total motor function assessment. Moreover, the Frontal Assessment Battery (FAB)^42^ was used to evaluate executive function, which involves performing a set of short mental and motor tasks.

### Statistical analysis

We tabulated the characteristics and results of all included studies. The statistical heterogeneity was also tested by *I*^2^, when *I*^2^ < 25% was identified as low heterogeneity^43^. We used a random effects model for heterogeneity because we assumed that there would be heterogeneity between studies using the *P* value (*P* < 0.05) and *I*^2^ statistic (*I*^2^ > 50%). All instruments were continuous variables, and we analyzed the SMD in the change from baseline and the 95% confidence interval (CI) for analysis. For studies that reported multiple interventions and comparators, we defined them as substudies to avoid double-counting and data neglect, and results from crossover studies were included in additional analyses (defined as sub-studies). We used StataMP statistical software (version 14, Stata Co. College Station, TX, United States) for meta-analysis.

## Additional Information

**How to cite this article**: Zhang, Y. *et al*. Improvement in Stroke-induced Motor Dysfunction by Music-supported Therapy: A Systematic Review and Meta-analysis. *Sci. Rep.* **6**, 38521; doi: 10.1038/srep38521 (2016).

**Publisher's note:** Springer Nature remains neutral with regard to jurisdictional claims in published maps and institutional affiliations.

## Supplementary Material

Supplementary Tables

## Figures and Tables

**Table 1:** Overall data of tests and baseline characteristics of included studies.

	Motor function tests	Studies (Analyses)	Participants (Intervention:control)	Age (SMD)	Gender (RR)	Stroke type (Hemorrhage/Ischemia,RR)	Position (Left/Right,RR)	Time post stroke (SMD)	trail design (RCT:CCT:RCT/crossover)	Delivery (Hospital:Rehabilitation centers)	Comparator (Active:usual care)	Quality (low/high)
Upper limb motor function	9-Hole Peg Test (9HPT,Pegs-minutes)^15^,^17^,^19^,^21^	4 (5)	172 (83:89)	0.28 (−0.13,0.70)	0.73 (0.56, 0.95)*	0.83 (0.39, 1.8)	1.10 (0.73,1.65)	0.87 (−1.33, 3.07)	1:2:1	4:0	2:3	3:1
	Box and Block Test (BBT,blocks/min)^15^,^19^,^21^	4 (4)	138 (64:74)	0.29 (−0.09, 0.67)	0.69 (0.52,0.93)*	1.30 (0.50, 3.37)	1.06 (0.65, 1.72)	—	0:2:1	3:0	2:2	2:1
	Arm Paresis Score (APS)^19^,^21^	2 (2)	102 (52:50)	0.28 (−0.11, 0.67)	0.69 (0.52,0.93)*	1.92 (0.71, 5.23)	1.06 (0.65, 1.72)	—	0:2:0	2:0	0:2	2:0
	Action Research Arm Test (ARAT)^19^,^21^	2 (2)	102 (52:50)	0.28 (−0.11, 0.67)	0.69 (0.52,0.93)*	1.92 (0.71, 5.23)	1.06 (0.65, 1.72)	—	0:2:0	2:0	0:2	2:0
Total motor function	Berg Balance Scale (score)^14^,^17^,^22^	2 (3)	73 (36:37)	0.20 (−0.26, 0.66)	0.93 (0.63, 1.39)	2.65 (0.97, 7.27)	1.13 (0.79, 1.61)	−0.34 (−0.81, 0.12)	2:0:0	1:1	2:0	0:2
	Fugl-Meyer assessment (FMA, score)^15^,^18^	2 (3)	66 (27:39)	—	—	—	—	—	1:0:1	1:1	2:1	0:2
	Wolf motor function test (score)^15^,^18^	2 (3)	66 (27:39)	—	—	—	—	—	1:0:1	1:1	2:1	0:2
	Wolf motor function test (time)^15^,^18^	2 (3)	66 (27:39)	—	—	—	—	—	1:0:1	1:1	2:1	0:2
	Stride length (cm)^14^,^23^	2 (3)	43 (21:22)	−0.20 (−0.84, 0.40)	—	—	1.18 (0.82, 1.70)	0.94 (0.14, 1.75)	2:0:0	1:1	0:2	0:2
	Gait Velocity (cm/s)^14^,^23^	2 (2)	43 (21:22)	−0.20 (−0.84, 0.40)	—	—	1.18 (0.82, 1.70)	0.94 (0.14, 1.75)	2:0:0	1:1	0:2	0:2
Executive functions	Frontal Assessment Battery (FAB, score)^16^,^20^	2 (8)	84 (39:45)	−0.12 (−0.49, 0.25)	0.97 (0.67, 1.38)	—	—	—	2:0:0	2:0	2:2	0:2

*Results with significant differences.
