# Supplementary material for: Improvement in Stroke-induced Motor Dysfunction by Music-supported Therapy: A Systematic Review and Meta-analysis
Source: Sci Rep. 2016 Dec 5;6:38521. doi: 10.1038/srep38521 (PMC5137001; doi:10.1038/srep38521)
Supplement: Supplementary Tables [file srep38521-s1.doc]

# Improvement in Stroke-induced Motor Dysfunction by Music-supported Therapy:

# A Systematic Review and Meta-analysis

Yingshi Zhang, PhD^1,2^; Jiayi Cai, PhD^1,2^; Yaqiong Zhang, PhD^1,2^; Tanshu Ren, MD^2^ ; Mingyi Zhao, PhD^1^; Qingchun Zhao, PhD^1,2*^

^1^School of Life Sciences and Biopharmaceutics, Shenyang Pharmaceutical University, Shenyang, 110016, P.R. China

^2^Department of Pharmacy, General Hospital of Shenyang Military Area Command, Shenyang, 110840, P.R. China

**^*^ The First Corresponding Author**

**Qingchun Zhao**

School of Life Sciences and Biopharmaceutics, Shenyang Pharmaceutical University

No. 103 Wenhua Road, Shenyang, 110016, China.

Tel.: +86 024 2885 6205; fax: +86 024 2885 6205

E-mail: [z](mailto:lqyxm@hotmail.com)haoqingchun1967@163.com (Q. Zhao).

**^*^ The Second Corresponding Author**

**Mingyi Zhao**

School of Life Sciences and Biopharmaceutics, Shenyang Pharmaceutical University

No. 103 Wenhua Road, Shenyang, 110016, China.

Tel.: +86 024 2398 6255; fax: +86 024 2398 6255

E-mail: zmy_dl@126.com (M. Zhao).

**Table S1 Study characteristics**

| **Publication, year, country** | **Subjects** | | | | | **Design** | | | | |
| --- | --- | --- | --- | --- | --- | --- | --- | --- | --- | --- |
|  | **Study design** | **Participants(N,age,male%)** | **Stroke type (Haemorrhage/Ischemia)** | **Time post stroke** | **Position(left/right)** | **Delivery** | **Intervention; music type** | **Intervention/Control(minutes/per week/weeks)** | **Assessment time** | **Outcomes** |
| van Vugt FT, 2016, Germany^15^ | RCT | I:19, 30-75, 52.63%  C:15, 30-75, 60% | I:0/19; C:6/9 | I:33.5±18.3(d)  C:39.2±28.7(d) | I:9/10  C:6/9 | Hospital | Interactive(piano training) | I: Music-supported therapy(90/3-4/10times)  C:No music care | 2-3w | 9HPT; BBS |
| Tong Y, 2015, China^16^ | RCT | I:15, 50.01±14.8, 86.67%  C:15, 48.6±14.6, 86.67% | - | I:5.4±4.8(m)  C:5.3±3.1(m) | I:8/7  C:6/9 | Rehabilitation centers | Interactive(musical instruments) | I: Music-supported therapy(30/5/4)  C:No music care | 4w | FMA,WMFT,WMFT-time |
| Cha Y, 2014, Korea^17^ | RCT | I:10,59.8±11.7,60%  C:10,63.0±14.1,60% | 20/0 | I:14.5±5.5  C:14.7±5.4 | I:9/1  C:9/1 | Hospital | Interactive | I:rhythmic auditory stimulation (30/5/6)  C: intensive gait training | 6wk | BBS; SL;GV |
| Friedman N, 2014, USA^18^ | RCT/crossover | 12,57±30.5 | 4/6; 2 unsure | 34.6±32.5 | 4/8 | Hospital | Passive(music glove); | I:Music glove(45/3/2)  C(a):IsoTrainer(45/3/2)  C(b):Tabletop exercises(45/3/2) | 2wk | BBT;9HPT; FMA,WMFT,WMFT-time |
| Särkämö T, 2010, Finland^19^ | RCT | I(a):11, 61.2±7.5, 36.36%  I(b):9, 59.7±7.1, 22.22%  C:9, 61.2±10.2, 66.67% | 29/0 | 6 | 0/29 | Hospital | Passive(CD player) | I:listen to music(45-90/-/6mo)  C:Usual therapy | 3mo,6mo | FAB |
| Altenmüller E, 2009, Germany^20^ | CCT | I:32,55.7±12.3,50%  C:30,53±11.8,80% | I:6/26; C:3/27 | NA | I:17/15  C:15/15 | Hospital | Interactive | I:Music-supported therapy(30/5/3)  C:Usual therapy | 3wk | ARAT, APS, BBT, 9HPT |
| Särkämö T, 2008, Finland^21^ | RCT | I:19,59.1±9.6, 63.16%  C(a):19, 59.3±8.3, 47.37%  C(b):17, 61.5±8.0, 47.06% | 55/0 | 6 | 55/0 | Hospital | Passive(CD player) | I: listen to music(-/-/2mo)  C(a): Audio book  C(b): Usual therapy | 3mo,6mo | FAB |
| Schneider S,2007,Germany^22^ | CCT | I:20,58.1±9.9,60%  C:20,54.5±10.2,75% | I:4/16  C:2/18 | I:2.1(m)  C:1.9(m) | I:1/18(unsure 1)  C:1/18(unsure 1) | Hospital | Interactive | I:Music-supported therapy(30/5/3)  C:Usual therapy | 3wk | ARAT, APS, BBT, 9HPT |
| van Nes IJ, 2006, Netherlands^23^ | RCT | I:26,62.6±7.6, 14/12  C:27,59.7±12.3,16/11 | I:11/16  C:4/22 | I:34.2 ±11.1d  C:38.9±9.2d | I:14/13  C:11/15 | Rehabilitation centers | Interactive | I:music therapy (4×45-second/5/6)  C:whole-body vibration | 6wk,12wk | BBS |
| Schauer M, 2003, Germany^24^ | RCT | I:11,59±12  C:12,61±12 | 23/0 | I:53d  C:47d | I:7/4  C:5/7 | NA | Interactive | I:musical motor feedback(20/5/3)  C:Usual therapy | 3wk | SL; GV |

I, intervention group; C, control group; RCT, randomized controlled trails; CCT, controlled clinical trials; RCT/crossover, randomized crossover trails;

NA, not application; 9HPT,9-Hole Peg Text(Pegs-minute);BBS,Berg Balance Scale(score); BBT,Box and Block Test (blocks/min); FAB, Frontal Assessment Battery

FMA, Fugl-Meyer assessment(score); GV,gait velocity(cm/s); SL,Stride length(cm); SL,Stride length(cm); WMFT,Wolf motor function test.

**Table S2 Methodological quality assessment by PEDro scale score**

| **Publication(year)** | I | II | III | IV | V | VI | VII | VIII | IX | X | XI | **Summary** |
| --- | --- | --- | --- | --- | --- | --- | --- | --- | --- | --- | --- | --- |
| van Vugt FT(2016)^15^ | Yes | 1 | 1 | 1 | 1 | 0 | 0 | 0 | 0 | 1 | 1 | 6 |
| Tong Y(2015)^16^ | Yes | 1 | 0 | 1 | 0 | 0 | 0 | 0 | 0 | 1 | 1 | 4 |
| Cha Y(2014)^17^ | Yes | 1 | 1 | 1 | 0 | 0 | 0 | 0 | 0 | 1 | 1 | 5 |
| Friedman N(2014)^18^ | Yes | 1 | 0 | 1 | 1 | 1 | 0 | 0 | 0 | 1 | 1 | 6 |
| Särkämö T(2010)^19^ | Yes | 1 | 1 | 1 | 0 | 0 | 0 | 1 | 0 | 1 | 1 | 6 |
| Altenmüller E(2009)^20^ | Yes | 0 | 0 | 1 | 0 | 0 | 0 | 0 | 0 | 1 | 1 | 3 |
| Särkämö T(2008)^21^ | Yes | 1 | 1 | 1 | 1 | 0 | 0 | 1 | 0 | 1 | 1 | 7 |
| Schneider S(2007)^22^ | Yes | 0 | 0 | 1 | 0 | 0 | 0 | 0 | 0 | 1 | 1 | 3 |
| van Nes IJ(2006)^23^ | Yes | 1 | 0 | 1 | 1 | 0 | 1 | 1 | 1 | 1 | 1 | 8 |
| Schauer M(2003)^24^ | Yes | 1 | 1 | 1 | 0 | 0 | 0 | 0 | 0 | 1 | 1 | 5 |

0, indicates the criterion was not satisfied; 1,the criterion was satisfied

I, Eligibility criteria specified(Yes/No); II, Random allocation(0/1); III, Concealed allocation(0/1); IV, Comparable at baseline(0/1); V,Blinded subjects(0/1); VI, Blinded therapists(0/1);VII, Blinded assessors(0/1); VIII,Adequate follow-up(0/1); IX, Intention-to-treat analysis(0/1); X, Between group comparisons(0/1); XI, Point estimates and variability(0/1)
